# Research progress of absorbable stents

**DOI:** 10.7150/ijms.90012

**Published:** 2024-01-01

**Authors:** Ying Song, Bingwei Li, Hao Chen, Zhuyuan Yu

**Affiliations:** 1 Department of Neurovascular oncology Surgery, First Hospital of Jilin University, 1 Xinmin Avenue Changchun 130021, Jilin Province, China.; 2 Department of Neurovascular Surgery, First Hospital of Jilin University, 1 Xinmin Avenue Changchun 130021, Jilin Province, China.

**Keywords:** Atherosclerosis, Bioresorbable stents, coating, Stent optimization

## Abstract

Atherosclerosis, a chronic inflammation of blood vessel walls, is a progressive pathophysiological process characterized by lipid deposition and innate adaptive immune responses. Arteriosclerosis often leads to narrowing of blood vessels. At present, interventional stent therapy is the main treatment method for vascular stenosis, which has the advantages of less trauma, less risk and faster recovery. However, atherosclerosis occurs in a complex pathophysiological environment. Stenting inevitably causes local tissue damage, leading to complications such as inflammation, intimal hyperplasia, late thrombosis, stent restenosis and other complications. It is urgent to optimize interventional therapy program. This article summarizes the advantages and disadvantages of absorbable metal scaffolds and the research progress of absorbable polymer scaffolds. The optimization strategy of stent is proposed. The status quo of drug coating was summarized. The prospect of new stent. To improve the therapeutic effect of arteriosclerosis.

## 1. Introduction

Atherosclerosis, the chronic inflammation of blood vessel walls, is a progressive pathophysiological process characterised by lipid deposition and innate adaptive immune responses. In response to blood flow disturbances, endothelial cells shift from a resting phenotype to a pro-atherosclerotic phenotype, which is often described as the starting point of atherosclerosis. It also causes excessive activation of the oxidative system, thrombosis, foam cell formation, inflammatory release, and sensitisation of SMC 1, gradually forming arteriosclerotic plaques and leading to stroke, coronary heart disease, and other diseases. It is a major cause of death in developed countries 2, 3.

Currently, interventional stenting is the most commonly used treatment for atherosclerotic stenosis. This method has the advantages of low trauma, low risk, and fast recovery and is clinically effective 4-6. However, atherosclerosis is characterised by a complex pathophysiological environment that includes low pH, high oxidative stress, and chronic inflammation. Stenting therapy inevitably causes local tissue damage, leading to complications such as inflammation, intimal hyperplasia, late thrombosis, and intrastent restenosis 7-9. Therefore, researchers continue to explore whether the implanted stent not only inhibits thrombus formation and promotes endothelialisation in the early stage but also inhibits cell proliferation in the later stage of treatment. Therefore, new stent technologies are being continuously developed. In this paper, we summarize the current research progress of absorbable stents and propose a scheme for material optimization. At the same time, the development of coating technology has also increased the effect of stent therapy. There are great expectations for bioabsorbable scaffolds.

## 2. Absorbable stent

### 2.1. Degradable metal stent

In recent decades, the use of bare-metal stents in clinical practice has improved the efficacy of arterial interventions 10, 11. However, the long-term presence of traditional permanent metal stents in patients can cause chronic inflammation, leading to intrastent restenosis and stent thrombosis 12. Moreover, long-term dual antiplatelet therapy after stent implantation may increase the risk of bleeding 13. Developing and improving a new generation of absorbable metal scaffolds (AMS) can replace permanent scaffolds and mitigate the associated risks. Ideal AMS can induce an appropriate host reaction and gradual corrosion, and its material and degradation products have sufficient safety and suitable biocompatibility 14-17 and can provide adequate mechanical integrity with appropriate elastic modulus, radial support strength, and ductility 18-20. Recently, magnesium, iron, and zinc, essential elements of the human body, have been considered candidates for making AMS. Stents made of these three materials are safe for implantation in the body 15, 20, 21. In addition, each of these three metals has unique characteristics. Magnesium stents release negligible amounts of magnesium ion (Mg^2+^) during degradation, which may inhibit abnormal nerve excitation and reduce the risk of atherosclerosis 22. Iron does not cause an excessive release of H^+^ or a sharp increase in pH; therefore, it has little impact on the local microenvironment 23. Zinc plays an important role in cell proliferation 20 and has demonstrated potential antibacterial and anti-atherosclerotic effects 24, 25.

However, in studies on biodegradable metal stents, differences in mechanical properties *in vivo* have limited their development. The radial strength and degradation rate of magnesium-based scaffolds are extremely high, and a large amount of H_2_ is generated during the degradation process, which creates an acidic environment in the local part of the scaffold and increases the corrosion rate 26. In addition, the mechanical properties of the support are reduced during corrosion. However, the degradation rate of iron-based scaffolds is extremely slow, resulting in the accumulation of corrosion products 15 (mainly iron oxide [Fe-O]) that remain in the encased neointima and inhibit vascular tissue regeneration 27. In addition, the magnetic properties of iron cannot be detected using magnetic resonance imaging 28. Moreover, the direct interaction between blood vessel cells and iron can generate harmful free radicals during corrosion 29, which can promote the oxidation and modification of nucleic acids and proteins, leading to oxidative stress and a range of harmful systemic events, such as ischaemia, inflammation, and neurodegeneration 30. In addition, the mechanical strength of zinc-based stents is insufficient, and alloying also leads to ductility and low strength of the zinc-based alloy 31.

### 2.2. Degradable polymer scaffold

Polylactic acid (PLA) is widely used in many biomedical applications owing to its biocompatibility, biodegradability, and nontoxic degradation products, especially in the fields of biodegradable stents and drug-carrying coatings. PLA, or polylactide, is a thermoplastic polyester with the main chain formula (C3H4O2)n, which is formed by the dehydration and condensation of lactic acid C(CH3)(OH)HCOOH. PLA has become a popular material; several different forms of polylactide exist: polyl-lactide (PLLA) is the product of the polymerisation of L, L-lactide 32, 33, poly (L-lactide-co-D, L-lactide) (PLD LLA) is used as a PLDLLA/TCP scaffold for bone engineering 34; poly (lactic acid-co-glycolic acid) (PLGA) is produced by random polymerisation of lactic acid and glycolic acid. PLGA is a functional, high-molecular-weight, degradable organic compound. PLA has good biocompatibility, non-toxicity, good encapsulation, and film-forming properties and is widely used in the pharmaceutical, medical engineering, and modern industrial fields 35, 36.

Biodegradable polymers are currently being studied in several clinical trials, including Absorb BVS (PLLA), Elixir Medical's DESolve (PLLA) stent, ART (PLDA) stent, and REVA's Fantom (PTD-PC) stent. These stents improve allergic reactions, atherosclerosis progression, and impaired vasomotor function after implantation of other stents 37.

However, some challenges exist in research on absorbable polymer scaffolds 38, 39, such as the mechanical properties of the device (thicker strut, lower radial strength, higher fracture sensitivity), longer than expected absorption time during absorption, stent removal due to uncovered strut discontinuity, demanding implantation procedures, high stent delivery failure rates, and stent expansion and misalignment. The incidence of acute and late stent thrombosis has increased 40.

Absorbable stents provide hope for treating vascular diseases by reducing the duration of oral dual antibody drugs and solving chronic inflammation caused by the long-term retention of stents in the body. However, metal scaffolds and polymer-absorbable scaffolds have mechanical properties such as degradation of metal scaffolds, corrosion rate, and radial strength of absorbable polymers. Therefore, new materials and designs are required to address this problem.

## 3. Optimisation of absorbable stent

### 3.1. Optimisation of metal support

Researchers have attempted to improve the performance of metal scaffolds by exploring new alloys, surface modifications, and manufacturing strategies.

#### 3.1.1. Alloying

Alloying can increase the mechanical strength, plasticity, and corrosion resistance of metal scaffolds 41, 42. In addition, rare earth elements can significantly improve the mechanical properties and degradation behaviour of biodegradable metals, such as (Y, Nd, Ho, Dy, and Gd) 43. Some alloying elements have been demonstrated to improve the degradation behavior of magnesium alloys, such as zinc, aluminum, manganese, calcium, lithium, strontium, and tin 44-46. However, to improve the degradation rate and biocompatibility of iron-based scaffolds, additives should have lower electrochemical potential or be more valuable than iron 18. For example, palladium or platinum can improve the mechanical properties, increase the corrosion rate of iron, and stabilise iron in the austenitic form 47. Iron-gold and iron-silver alloys have faster degradation rates without increased cytotoxicity, platelet adhesion, or thrombogenic effects 48. Iron nitride exhibits a high degradation rate and good mechanical properties 49. The mechanical strength of zinc is insufficient for stent implantation; however, the zinc-based scaffold is optimised for its degradation rate and biocompatible, mainly through alloying, to improve tensile strength 31. For example, copper, magnesium, calcium, and strontium can further improve the mechanical properties and degradation of zinc-aluminum alloys 50. Zinc-silver alloys can reduce stent-associated infections and adjust mechanical strength by adjusting the silver content 51.

#### 3.1.2. Surface modification

Micro-arc oxidation, phosphating treatment, electrodeposition, and basic heat treatment can change the surface chemistry and metallurgical microstructure of magnesium-based scaffolds and improve the degradation behaviour of magnesium scaffolds 52. Using plasma immersion ion implantation and deposition, Fe-O films can be constructed to cover iron-based scaffolds, thereby improving the biocompatibility and mechanical activation of platelets 53. Surface modifications can help improve the corrosion rate of iron-based scaffolders, such as lithography and electron beam evaporation of platinum disks, sandblasting, phosphating, alkaline heating, micro-arc oxidation, and electrodeposition 54. However, these technologies need to be explored further.

#### 3.1.3. Improving the manufacturing strategy

New manufacturing strategies to achieve grain refinement can also improve the mechanical properties and degradation behaviour of magnesium-based scaffolds. For example, AZ31 exhibits a lower degradation rate due to grain refinement from mechanical processing 55. The small ZM21 has a higher mechanical strength 56. In addition, 3D printing technology, particularly selective laser melting, can be used to process magnesium alloys to optimise the machine structure and better control corrosion 57, 58. Equal-channel angular pressure can produce nanocrystalline iron, which inhibits VSCM proliferation but promotes ECs growth 59. Owing to micrograin and microstructural defects, electroforming processes increase the degradation rate of iron-based scaffolds, resulting in the increased release of iron 60. Simultaneously, iron-based scaffolds produced by powder metallurgy (PM) have faster corrosion rates because PM creates more pores 61. Several new manufacturing methods, including inkjet 3D printing and power spraying of cold air 62, may also improve the degradation rate of iron-based scaffolds. In addition, the mechanical properties of cast zinc alloys can be further improved by grain refinement induced by deformation heat treatment 63. Severe plastic deformation techniques may alter the mechanical properties of zinc alloys 64.

### 3.2. Optimisation of polymer scaffolds

Various methods have proved effective for strengthening absorbable polymer scaffolds, including fabrication techniques, geometric parameter optimisation, and scaffold thickness enhancement 65. High molecular weight polymers can increase the entanglement and length of covalently bonded molecular chains, thereby improving the fracture strain and wear resistance of scaffolds 66. Increasing the crystallinity of semi-crystalline polymers can increase the hardness and heat or chemical resistance of BDPS 67. Modifying the molecular structure by controlling the internal structure of the polymer chain orientation can improve the mechanical strength of the scaffold 68. Simultaneously, changing the geometric parameters can improve the radial strength of the support. For example, the IGaki-Tamai stent has a thick strut in the shape of a zigzag spiral coil; therefore, it has high vascular coverage 69. Biodegradable, nontoxic lignocellulosic fibres from renewable resources such as wood have been studied as potential augments for biodegradable polymers because of their high strength and more economical performance than traditional synthetic fibers 70-73. In addition, fabricating absorbable polymer scaffolds with CO_2_ lasers or adopting new sliding lock mechanisms can improve the mechanical properties of scaffolds 74. The shape memory PCLAU combined with Fe_3_O_4_ nanoparticles can provide sufficient strength for stent implantation 75. The biocompatibility of BDPS can be improved by plasma surface treatment and the use of high-molecular-weight PLLA 69. In addition, the degradation of BDPS can be improved by changing the crystallinity, molecular weight, and hydrophilicity of the polymer 76-78.

## 4. Stent coating

Coating technologies for biomaterials include metal-metal coating, chemical vapour deposition, ion beam assisted deposition, atomic layer deposition, and pulsed laser deposition 79-81. Using these technologies, targeted drugs, bio-based coatings, polymer coatings, and inorganic coatings can be delivered to the target location. Polymer coatings and inorganic coatings can also be used to produce porous coatings.

### 4.1. Drug coating

In recent years, research has been increasingly conducted on new drugs for drug-eluting stents. Currently, drug-eluting stents commonly used in clinics mainly use drugs such as rapamycin, paclitaxel, sirolimus, and everolimus to inhibit endothelial and smooth muscle proliferation, and their short-term therapeutic effects have been confirmed. However, the incidence of long-term stent thrombosis and restenosis remains a challenge 82-84. Therefore, scientists are constantly exploring new drugs and drug combinations to optimise drug coatings. Heparin induces and accelerates endothelial cell regeneration and maintains anticoagulant activity 85. Statin drug-eluting stents eliminate atherosclerotic plaque 86. They induce autophagy at atherosclerotic sites and exert anti-inflammatory effects 87. In addition to synthetic drugs, gene mediators are also of interest because they integrate smoothly during physiological regulation. Nitric oxide (NO) is an endogenous gas signalling molecule that regulates vasodilation, controls smooth muscle cell proliferation, inhibits platelet aggregation, and has antibacterial and anti-inflammatory functions 88. Researchers have prepared a catalyst on the scaffold 89 that catalyses the release of NO to improve anticoagulation and prevent scaffold restenosis. H_2_S is another gas-signalling molecule that plays an important role in maintaining cerebrovascular homeostasis and protecting and regulating the central nervous system. H_2_S promotes angiogenesis and anti-inflammatory mediators 90-93. The aspirin derivative ACS14 and its metabolite ADTOH are potential H_2_S donors 94. ACS14 releases H_2_S while maintaining the antithrombotic effects of aspirin.

### 4.2. Bio-based coatings

Special biological material-based scaffold coatings are desirable. They allow endothelial cells on scaffold surfaces to proliferate, differentiate, release, and grow, inhibit thrombosis and neointimal hyperplasia, and alleviate restenosis. Endoglin antiboil-coated scaffolds significantly reduced restenosis by enhancing reendothelialisation in pig models 95. Coating with anti-CD146 antibody (Ab) -fixed silicon nanofilaments for the efficient and specific capture of late rather than early EPCs demonstrated an approximately two-fold increase in endothelial coverage 96. In addition, endothelial progenitor cell-capture scaffolds with surface-immobilised antibodies have demonstrated clinically significant improvements in endothelialisation. However, most current antibody-based scaffold surface modification strategies rely on antibody adsorption or direct coupling via amino or carboxyl groups, which results in poor control of the antibody surface concentration and/or molecular orientation and eventual cell capture bioavailability. Cell capture is enhanced by the covalent transplantation of protein G polypeptides to immobilise IgG antibodies 97. The effect of angiogenic factors (VEGF) on endothelial regeneration and the prevention of restenosis after stenting is expected, especially when combined with a drug-eluting coating, which exhibits significant endothelial regeneration and maintains a very low level of intrastent restenosis 98. In addition, cell coating is promising, and stents coated with VEGF/HGF-secreting UCB-MSCs reduce the restenosis side effects of cardiac stent implantation and improve reendothelialisation 99.

### 4.3. Polymer coating

Polymer materials have been used as stent coatings with or without drug elution with mixed success rates. These materials include polyethylene, polyurethane, polyvinyl ester, and polylactide. They can be used as nanomaterials and drug carriers to control drug release rates 14, 100-103. However, frequently used biodegradable synthetic polymers such as PLGA and PLLA produce acidic degradation products that cause local inflammation and delay tissue healing due to local acidification 104, 105. Naturally derived biopolymers, such as zein (from corn) and alginate (from seaweed), have been used to replace synthetic biodegradable polymers with less inflammation in long-term applications 106.

### 4.4. Inorganic coatings

Several inorganic materials can potentially improve the performance of implant surfaces. The inorganic materials used to manufacture scaffold coatings include oxides, nitrides, silicides and carbides, precious metals, hydroxyapatite-based materials, and diamond and diamond-like carbon 107-110. Titanium oxide-based coatings are the most promising inorganic materials for cardiovascular stents. The stainless steel bioactive scaffold Titan2 (Hexacath, Paris, France), coated with plasma-enhanced titanium vapour deposition in a nitrogen-oxygen mixed atmosphere, inhibits platelet aggregation, minimises fibrin deposition, reduces inflammation, and promotes healing. In recent clinical trials 111-113, the new generation of titanium NO-coated stents, TiOxNy and TITAX-AMI, have been proven safe, successfully reduced in-stent restenosis, and marketed. NO is one of the most important molecules in biological systems and plays a key role in pathophysiology and disease by promoting endothelialisation and activating endothelial cell growth. This has led to the development of novel therapeutic strategies and NO donors 114.

## 5. Outlook

Given the high mortality rates worldwide from cardiovascular and cerebrovascular diseases caused by arteriosclerosis and the potential of stent technology, researchers and clinicians are focused on developing new materials, methods, and solutions to improve clinical outcomes for the available types of stents. The goal is patient safety and to achieve a higher success rate for cardiovascular therapy. Developing and optimising new categories of scaffolds, including membrane-coated and bioabsorbable scaffolds, can achieve the desired release of bioactive agents for adhesion, cell differentiation, and tissue development with appropriate physicochemical properties and degradation rates. Stents for personalised treatment are expected to become available in the near future.

## 6. Conclusion

Cardiovascular and cerebrovascular diseases caused by atherosclerosis have high mortality rates in developed countries. Interventional stenting is the most common treatment for atherosclerosis. This method involves less trauma, less risk, and faster recovery, and is clinically effective. Shortcomings exist in the current study of stents; however, the continuous exploration of new stent materials and the optimisation of structural design, the continuous development of reasonable drug release and biotechnology, the realisation of targeted therapy for arteriosclerosis, and the concept of intervention-free implantation are needed.

## Figures and Tables

**Table 1:** The main preclinical research of stent in arteriosclerosis

	Materials	Coating	Drugs	Outcome
105	316L stainless steel	Hyaluronic acid and chitosan	ACS14	Inhibit platelet adhesion and activation Inhibition of smooth muscle cells and macrophages proliferation Reduced inflammation at the site of intervention and promoted the formation of new blood vessels
115	316L stainless steel	PLCL	Atorvastatin Fenofibrate	Excellent biocompatibility No inflammatory reaction.
104	316L stainless steel	zein (the active layer) and cross-linked alginate (the sacrificial layer)	rutin	Sustained drug release High biocompatibility
116	316L stainless steel	PLLA	SZ-21、VEGF、RAPA	Reendothelialization and inhibition of thrombosis, inflammation, and intrastent restenosis
85	316L stainless steel	PGMA	Hep/NONOates nanoparticle	Endothelial cell regeneration Anticoagulant activity
86	316L stainless steel	EGCG	Pivastatin calcium	Reactive oxygen species
117	316L stainless steel	Avidin	biotin-modified endothelial cells	Promotes cell bonding to scaffold struts Reduce intrastent restenosis
118	316L stainless steel	PDA-HD	Multifunctional coating of flavonoids baicalin	Anti-ISR, anti-inflammation Promote endothelialization
119	cobalt-chromium alloy	Polylysine layer and hyaluronic acid-dopamine conjugate	NO	Optimize the release rate and therapeutic dose of NO
120	cobalt-chromium alloy	Hyaluronic acid/chitosan	Binding siRNA nanocomplexes	Good blood compatibility
96	cobalt-chromium alloy	silicone nanofilament (SiNf)	CD146- Antibody	Promote reendothelialization Preventive restenosis
121	Mg-Zn alloy	TiO2	TiO2 nanocoating	Stimulate endothelial cell adhesion and proliferation Inhibit the release of harmful products from zinc-magnesium coated scaffolds
122	Mg-Zn alloy	MF2-PA-PLGA	Rare-earth free	Complete biodegradation No foreign body residue Promote reendothelialization
123	Fe base	PDLLA	Sirolimus	Promote reendothelialization Reasonable corrosion
124	Zn base	Mixed coating of polycarbonate, tannic acid and copper ions	Copper	Corrosion resistance Reduces inflammation Promote endothelial cell adhesion and proliferation
125	PLLA	PLLA	4-octyl itaconate (OI)	Decreased inflammation Inhibition of SMC proliferation Endothelial regeneration integrity
98	PLLA	PLGA	Rapamycin, VEGF	Promotes endothelial regeneration Reduce intrastent restenosis
126	PLLA	PCL-PEG-PCL, PCEC	miR-22	Reduce inflammation Phenotypic transformation of SMC was low IRS suppression
106	PU	zein	ZnO nanoparticles	Cytocompatibility Anticoagulant reaction Antibiosis

## Abbreviations

PLA: Polylactic acid
PLLA: polyl-lactide
PLDLLA: poly (L-lactide-co-D, L-lactide)
PLGA: poly (lactic acid-co-glycolic acid)
PM: powder metallurgy
NO: nitric oxide
